# Sarcoma protein kinase inhibition alleviates liver fibrosis by promoting hepatic stellate cells ferroptosis

**DOI:** 10.1515/biol-2022-0781

**Published:** 2023-12-06

**Authors:** Zhengyuan Cheng, Xiaojuan Zhang, Pingsheng Chen, Haitao Wang, Kuangjing Wang, Yingzhou Shen

**Affiliations:** Department of Internal Medicine (Gastroenterology), Maanshan Clinical School of Anhui Medical University (Maanshan People’s Hospital), Hubei Road 45, Huashan District, Maanshan 243099, Anhui Province, China; Department of Nephrology, Jinling Hospital Affiliated to Nanjing University, Zhongshan East Road 305, Xuanwu District, Nanjing 210008, Jiangsu Province, China; Department of Pathology and Pathophysiology, Medical School, Southeast University, Dingjiaqiao 87, Gulou District, Nanjing 210009, Jiangsu Province, China

**Keywords:** liver fibrosis, hypoxia, hepatic stellate cells, sarcoma protein kinase, ferroptosis

## Abstract

Liver fibrosis is a type of chronic pathological liver damage involving liver tissue hypoxia and abnormal extracellular matrix deposits. Hepatic stellate cells (HSCs) activation is critical for liver fibrosis. Currently, inhibiting HSCs activation or inducing HSCs ferroptosis is considered an effective strategy for the treatment of liver fibrosis. Sarcoma protein kinase (Src) is an important member of the tyrosine protein kinase family. Hypoxia causes Src phosphorylation at tyrosine 416 (Tyr 416), and inhibiting Src activation can alleviate liver fibrosis. There is currently little research on the relationship between Src activation and ferroptosis in liver fibrosis. 1-(1,1-Dimethylethyl)-1-(4-methylphenyl)-1*H*-pyrazolo[3,4-d]pyrimidin-4-amine (PP1) is an inhibitor of Src activation at Tyr 416. Therefore, in this study we treated HSC-T6 cells with PP1 under normoxic and hypoxic culture conditions; moreover, PP1 was also used to treat a carbon tetrachloride-induced mouse liver fibrosis model. We explored whether inhibiting Src activation could alleviate liver fibrosis by promoting HSCs ferroptosis *in vitro* and *in vivo*. *In vitro* experiments showed that inhibiting Src activation in HSC-T6 cells significantly reduced hypoxia-inducible factor-1α (HIF-1α) expression and HSC-T6 cells activation, and ferroptosis was significantly increased. *In vivo* experiments revealed that inhibiting Src activation in fibrotic livers reduced HIF-1α expression; meanwhile, ferroptosis was promoted, and liver fibrosis was alleviated. Therefore, inhibiting Src activation, which increases HSCs ferroptosis, can alleviate liver fibrosis.

## 1 Introduction

Liver fibrosis is the common developmental stage of many chronic liver diseases. If the liver tissue continues to be damaged, fibrosis will develop into cirrhosis, which leads to liver failure [1]. Numerous studies have confirmed that hepatic stellate cells (HSCs) activation is critical for the development of liver fibrosis. Normally, HSCs are in a quiescent state, and when the liver is continuously damaged, HSCs can be activated to form myofibroblasts (MFs), which is accompanied by high expression of α-smooth muscle actin (α-SMA), Vimentin, and connective tissue growth factor (CTGF) [2–4]. Activated HSCs can synthesize excessive levels of extracellular matrix (ECM), which is critical to the occurrence of liver fibrosis [5]. Ferroptosis is a newly identified iron-dependent form of programmed cell death that results from the accumulation of lipid peroxides. Kong et al. found that artesunate could inhibit the development of liver fibrosis by inducing ferroptosis in activated HSCs [6]. Another study has shown that the upregulation of heme-oxygenase-1 can induce ferroptosis in activated HSCs, thereby inhibiting liver fibrosis [7]. Thus, inducing ferroptosis in activated HSCs is an important strategy to treat liver fibrosis.

During liver fibrosis, hypoxic damage occurs in liver tissue, and hypoxia-inducible factor-1α (HIF-1α) is critical in hypoxic damage, which has been confirmed by many studies [8,9]. Furthermore, HIF-1α is an important factor in activating HSCs and promoting ECM production [10]. Sarcoma protein kinase (Src) is an important member of the tyrosine protein kinase family. Hypoxia can cause the phosphorylation of Src at tyrosine 416 (Tyr 416) [phospho-Src (Tyr 416)]. Phospho-Src (Tyr 416) can regulate a variety of signaling pathways, such as [11–13] HIF-1α, transforming growth factor (TGF-β)/Smad, and signal transducer and activator of transcription 3, and the activation of these pathways can promote the occurrence of fibrosis. Cheng et al. found that in the fibrotic renal tissues of rats, HIF-1α expression was significantly increased, and Src was activated at Tyr 416, which promoted the progression of renal fibrosis [13]. 1-(1,1-Dimethylethyl)-1-(4-methylphenyl)-1*H*-pyrazolo[3,4-d]pyrimidin-4-amine (PP1) is a small molecular compound that can inhibit the phosphorylation of Src at Tyr 416 and thus can play a therapeutic role in a variety of diseases, such as renal fibrosis, ischemic stroke, and pulmonary arterial hypertension [11,14,15]. In renal fibrosis, when PP1 is used to inhibit Src activation, it can reduce HIF-1α expression, which is one of the important mechanisms to alleviate renal fibrosis [13]. HIF-1α can promote HSCs activation; therefore, reducing HIF-1α expression in liver fibrosis plays an important role in alleviating liver fibrosis [16]. Unfortunately, there has been little research on whether inhibiting Src activation can also reduce HIF-1α expression in liver fibrosis. More importantly, it has been confirmed that promoting activated HSCs ferroptosis can alleviate liver fibrosis, but it is still unclear whether inhibiting Src activation at Tyr416 can regulate activated HSCs ferroptosis.

Therefore, in this study, the HSC-T6 cell line and a carbon tetrachloride (CCl_4_)-induced mouse liver fibrosis model was used to examine whether inhibiting Src activation could promote ferroptosis in HSCs, and whether liver fibrosis could be alleviated through this process. The results showed that inhibiting Src activation, which increased HSCs ferroptosis, could alleviate liver fibrosis. And these findings will provide new ideas for the treatment of liver fibrosis.

## 2 Materials and methods

### 2.1 Cell culture and cell counting kit-8 (CCK-8) cytotoxicity analysis

HSC-T6 cells were purchased from the Shanghai Cell Bank of the Chinese Academy of Sciences and cultured in high-glucose DMEM (Servicebio, Wuhan, China) with 10% fetal bovine serum (Tianhang, Zhejiang, China) at 37°C in a 5% CO_2_ incubator (Thermo, Waltham, USA). The CCK-8 cytotoxicity assay (Apexbio, Houston, USA) was used to determine the treatment time and dose of PP1 (Apexbio). PP1 was dissolved in dimethyl-sulfoxide (DMSO, Beyotime, Shanghai, China). Under hypoxic conditions, different concentrations of PP1 (0, 5, 10, 15, 20, and 25 μM) were administered to HSC-T6 cells, which were divided into six groups of 5 × 10^3^ cells/well in 96-well plates (NEST Biotechnology, Wuxi, China) with 100 µL of culture medium in each well. A hypoxic incubator (Thermo) was used to achieve a hypoxic environment (94% N_2_/5% CO_2_/1% O_2_).

After 24, 48, and 72 h, the optical density at 450 nm was measured by a microplate reader (Biotek, Winooski, USA). After the treatment dose (10 μM) and time (24 h) were determined, the cells were divided into four groups and cultured under different conditions. In the normoxia group, the cells were cultured in normoxic conditions without any treatment; in the hypoxia group, the cells were cultured in hypoxic conditions without any treatment; in the hypoxia + DMSO group, the cells were cultured in hypoxic conditions with DMSO; and in the hypoxia + PP1 group, the cells were cultured in hypoxic conditions with PP1.

### 2.2 Immunofluorescence analysis

The four groups of cells were cultured in 24-well plates, fixed with 4% paraformaldehyde and then washed with PBS. Then, the cells were incubated with primary antibodies against α-SMA (1:200, Proteintech, Rosemont, USA), Vimentin (1:200, Proteintech), and Collagen-I (1:200, Affinity Biosciences, Cincinnati, USA) overnight at 4°C. The next day, the cells were incubated with a Coralite-594-conjugated secondary antibody (1:400, Proteintech) at 37°C for 1 h. 4′,6-Diamidino-2-phenylindole (Servicebio) was used to stain the nuclei. Fluorescence microscopy (Olympus, Tokyo, Japan) was used to observe the results. The results were analyzed by ImageJ.

### 2.3 Animal model

Male institute of cancer research mice (ICR mice, 8 weeks old) were purchased from Qinglongshan Biotechnology Company (Nanjing, China) and housed in an air-, temperature-, and light-controlled facility. The mice were divided into two groups. One group was untreated and served as a blank control (control group, *n* = 15). The other group was the model group (*n* = 45), and a liver fibrosis model was constructed by intraperitoneal injections of CCl_4_ (Titan, Shanghai, China) as follows: a 20% CCl_4_ olive oil solution was administered at a dose of 5 mg/kg twice per week for 8 weeks. There were a total of nine deaths in the model group during the experimental period.

The control group did not change after 8 weeks. The model group was divided into three new groups (*n* = 12/group): one group was untreated (CCl_4_ group), one group was intraperitoneally injected with DMSO for 1 week (CCl_4_ + DMSO group, DMSO 1.6 ml/kg), and one group was intraperitoneally injected with DMSO containing PP1 for 1 week (CCl_4_ + PP1 group, PP1 5 mg/kg). No mice died during the experimental period. The mice were sacrificed after 1 week, body weight was recorded before sacrifice, and the livers were collected and weighed. The liver weight/body weight ratio was calculated, and the liver tissues were retained for further analysis.


**Ethical approval:** The research related to animal use has been complied with all the relevant national regulations and institutional policies for the care and use of animals, and was approved by animal ethics committee of Jinling Hospital affiliated to Nanjing university (No. 2019JLHGKJDWLS-161).

### 2.4 Histopathology

Mouse liver tissue was fixed with 10% formalin for 24 h, embedded in low-temperature paraffin, and cut into 3 μm-thick slices. Then, hematoxylin–eosin (HE) staining and Masson staining were used to examine the pathological changes in liver tissues. The fibrosis score of each group was calculated by the Scheuer scoring system [17].

### 2.5 Immunohistochemistry

First, the specimens were incubated with anti-α-SMA (1:200, Abcam, Cambridge, UK) and anti-Vimentin (1:2,500, Proteintech) primary antibodies at 4°C overnight, and each slide contained a negative control. Next, a one-step polymer detection kit (Maixin, Fuzhou, China) was used to visualize the results. Finally, the results were observed and evaluated.

### 2.6 Western blotting

HSC-T6 cells and fresh mouse liver tissues were collected and lysed to extract the proteins. The protein concentration was measured by a bicinchoninic acid protein assay kit (Beyotime). The protein levels of HIF-1α, Src, p-Src (Tyr416), CTGF, Collagen-I, SLC7A11, GPX4, P-53, β-actin, GAPDH, and HSP90 were measured by western blotting. Rabbit anti-HIF-1α (1:1,000, Abcam), rabbit anti-Collagen-I, CTGF (1:1,000, Affinity Biosciences), rabbit anti-Src, SLC7A11 (1:1,000, Proteintech), rabbit anti-P-53, HSP90 (1:5,000, Proteintech), rabbit anti-p-Src (Tyr416) (1:1,000, CST, Danvers, USA), rabbit anti-β-actin, GAPDH (1:2,000, Servicebio), and mouse anti-GPX4 (1:1,000, Proteintech) primary antibodies were used. Horseradish peroxidase-linked goat anti-rabbit (1:3,000, Servicebio) and goat anti-mouse (1:3,000, Servicebio) secondary antibodies were used. An enhanced chemiluminescence (ACE Biotechnology, Nanjing, China) imaging method was used to visualize the protein bands, and the results were analyzed by ImageJ software (Version 1.53, NIH, Bethesda, USA).

### 2.7 Ferroptosis detection *in vitro* and *in vivo*

#### 2.7.1 Reactive oxygen species (ROS) detection

The levels of ROS in cells were measured with a ROS detection kit (Beyotime). HSC-T6 cells were inoculated and treated in 24-well plates according to the conditions described in Section 2.1, washed with PBS, and then cultured in medium containing 10 μM 2,7-dichlorodi-hydrofluorescein diacetate for 30 min. The cells were observed with a fluorescence microscope (Olympus), and the results were analyzed with ImageJ.

#### 2.7.2 Reduced glutathione (GSH), malondialdehyde (MDA), and Fe^2+^ analysis

A GSH test kit (Boxbio, Beijing, China), MDA test kit (Beyotime), cell Fe^2+^ test kit (Elabscience, Wuhan, China), and animal tissue Fe^2+^ test kit (Elabscience) were used to determine the levels of corresponding substances in the four groups of cells and four groups of liver tissues. The specific steps were performed according to the instructions.

#### 2.7.3 Mitochondrial observations

HSC-T6 cells were cultured as described in Section 2.1 and then fixed, dehydrated, soaked, embedded, and stained with lead citrate. Mouse liver tissues were sliced, fixed, dehydrated, soaked, embedded, and stained with lead citrate. Finally, images were obtained using transmission electron microscopy (TEM, Hitachi TEM system, Tokyo, Japan).

#### 2.7.4 Western blotting

The protein levels of SLC7A11, P-53, and GPX4 were measured by western blotting as described in Section 2.6.

### 2.8 Statistical analysis

The data are expressed as the mean ± standard deviation for each group. Intergroup comparisons were made using one-way analysis of variance, and the differences between two groups were determined by Student’s *t* test (Graphpad prism v8.0, San Diego, USA). Statistically significant differences between different groups are marked in each graph. A value of *P* < 0.05 was considered significant. All experiments were repeated three times.

## 3 Results

### 3.1 Effect of inhibiting Src activation on HIF-1α expression and HSC-T6 cells activation

#### 3.1.1 CCK-8 cytotoxicity assay

**Figure 1:**
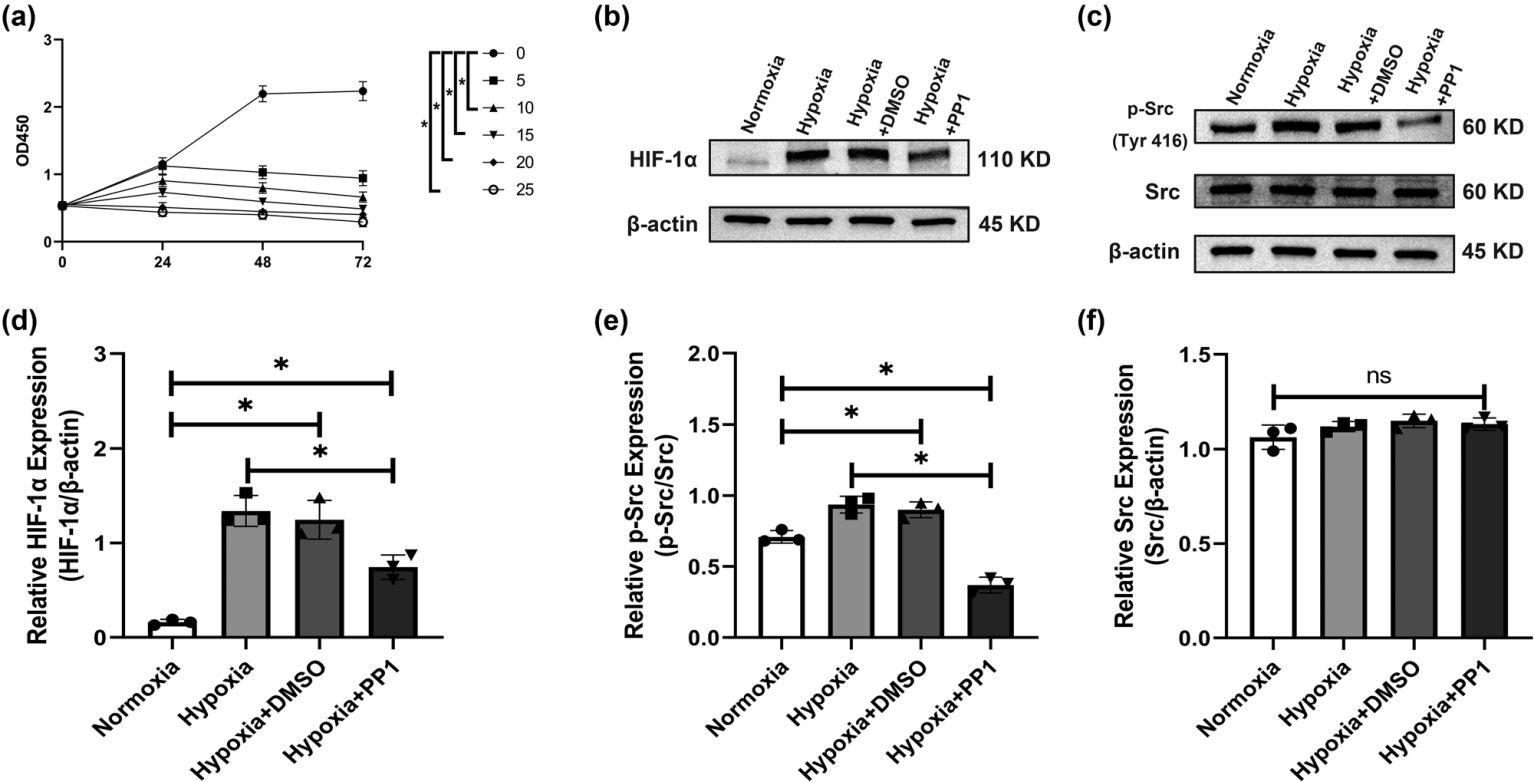
CCK-8 assay results and protein levels of HIF-1α, p-Src, and Src in the different groups. (a) CCK-8 OD450 results. *P < 0.05 compared with 0 μM. (b and c) Protein levels of HIF-1α, p-Src (Tyr 416), and Src. (d–f) Relative expression of HIF-1α, p-Src (Tyr 416), and Src. *P < 0.05 compared with the other groups.

After 24 h of hypoxic culture, cytotoxicity was observed in HSC-T6 cells that were treated with 10, 15, 20, and 25 μM PP1. After 48 and 72 h, cytotoxicity was observed in HSC-T6 cells that were treated with 5, 10, 15, 20, and 25 μM PP1 (Figure 1a).

#### 3.1.2 Protein levels of HIF-1α, p-Src (Tyr 416), and Src in HSC-T6 cells

Western blotting showed that the protein levels of HIF-1α and p-Src (Tyr416) were significantly lower in the normoxia group and hypoxia + PP1 group than in the hypoxia group and hypoxia + DMSO group (Figure 1b–e). No obvious change was observed in the protein level of Src among the four groups (Figure 1c and f).

#### 3.1.3 Inhibiting Src activation can reduce HSC-T6 cells activation

**Figure 2:**
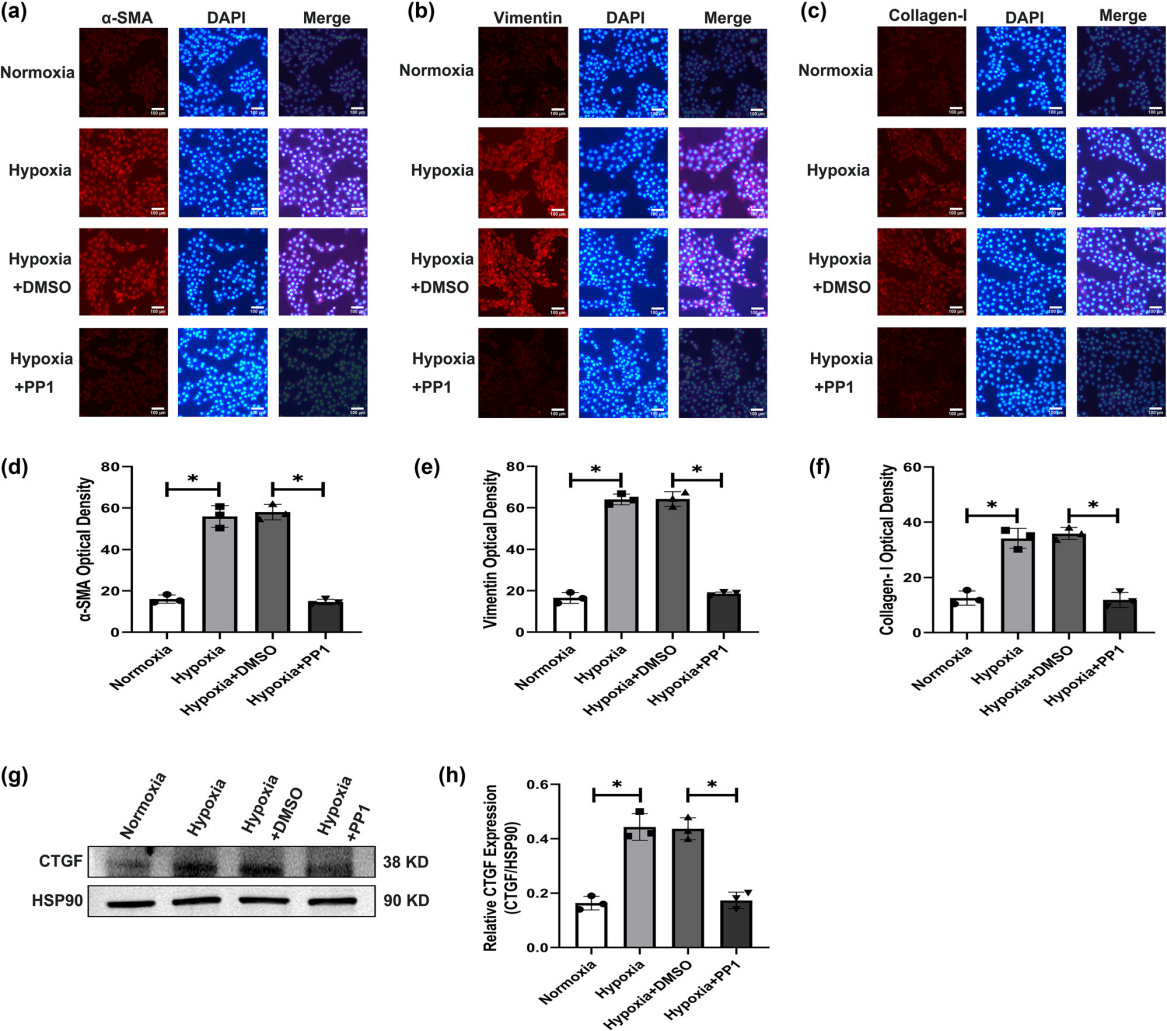
Expression of activation-related markers in HSC-T6 cells under different culture conditions. (a) α-SMA expression. (b) Vimentin expression. (c) Collagen-I expression. (d–f) Relative expression of α-SMA, Vimentin, and Collagen-I in each group, *P < 0.05. (g) Protein levels of CTGF. (h) Relative expression of CTGF in each group, *P < 0.05.

The immunofluorescence results showed that the expression levels of the activation indicators α-SMA, Vimentin, and Collagen-I in HSC-T6 cells were significantly lower in the normoxia group and hypoxia + PP1 group than in the hypoxia group and hypoxia + DMSO group (Figure 2a–f). Western blotting suggested that the protein level of CTGF was significantly lower in the normoxia group and hypoxia + PP1 group than in the hypoxia group and hypoxia + DMSO group (Figure 2g and h).

### 3.2 Inhibiting Src activation can promote ferroptosis in HSC-T6 cells

**Figure 3:**
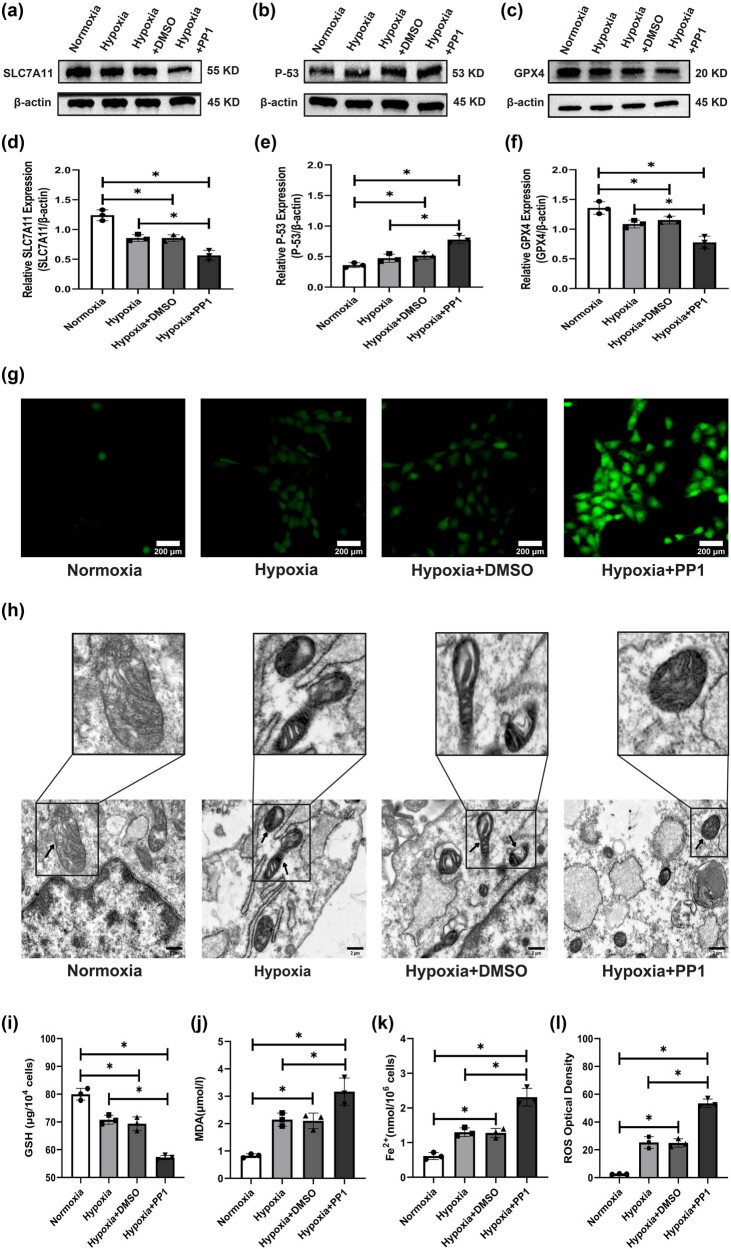
Protein levels of SLC7A11, P-53, and GPX4; ROS levels; mitochondrial morphology; and the levels of GSH, MDA, and Fe^2+^ in the different groups. (a–c) Protein levels of SLC7A11, P-53, and GPX4. (d–f) Relative expression levels of SLC7A11, P-53, and GPX4, *P < 0.05. (g) ROS levels in the different groups. (h) Mitochondrial morphology in the different groups; arrows denote normal mitochondria (control group) and shrunken mitochondria (other groups). (i–k) GSH, MDA, and Fe^2+^ levels in the different groups, *P < 0.05. (l) Relative levels of ROS in each group, *P < 0.05.

Western blotting showed that the protein levels of the ferroptosis genes SLC7A11 and GPX4 in HSC-T6 cells were significantly decreased in the hypoxia, hypoxia + DMSO, and hypoxia + PP1 groups compared with the normoxia group, and the expression levels of these two proteins were lowest in the hypoxia + PP1 group (Figure 3a, c, d, and f). However, the protein levels of the ferroptosis gene P-53 in HSC-T6 cells were significantly increased in the hypoxia, hypoxia + DMSO, and hypoxia + PP1 groups compared with the normoxia group, and the expression levels of this protein was highest in the hypoxia + PP1 group (Figure 3b and e). ROS analysis suggested significant ROS production in HSC-T6 cells in the hypoxia, hypoxia + DMSO, and hypoxia + PP1 groups compared to the normoxia group, and ROS levels in the hypoxia + PP1 group were the highest (Figure 3g and l). TEM indicated shrunken mitochondria in the hypoxia, hypoxia + DMSO, and hypoxia + PP1 groups, but in the normoxia group, mitochondrial morphology was normal (Figure 3h). GSH analysis suggested that GSH levels in the hypoxia, hypoxia + DMSO, and hypoxia + PP1 groups were lower than those in the normoxia group, and GSH levels in the hypoxia + PP1 group were the lowest (Figure 3i). However, compared with those in the normoxia group, the levels of MDA and Fe^2+^ in the hypoxia group, the hypoxia + DMSO group, and the hypoxia + PP1 group were increased and were highest in the hypoxia + PP1 group (Figure 3j and k).

The *in vitro* experiments showed that inhibiting Src activation not only inhibited the activation of HSC-T6 cells by reducing HIF-1α expression, but also promoted ferroptosis in HSC-T6 cells. Next, we performed *in vivo* experiments.

### 3.3 Therapeutic effects of inhibiting Src activation on liver fibrosis

**Figure 4:**
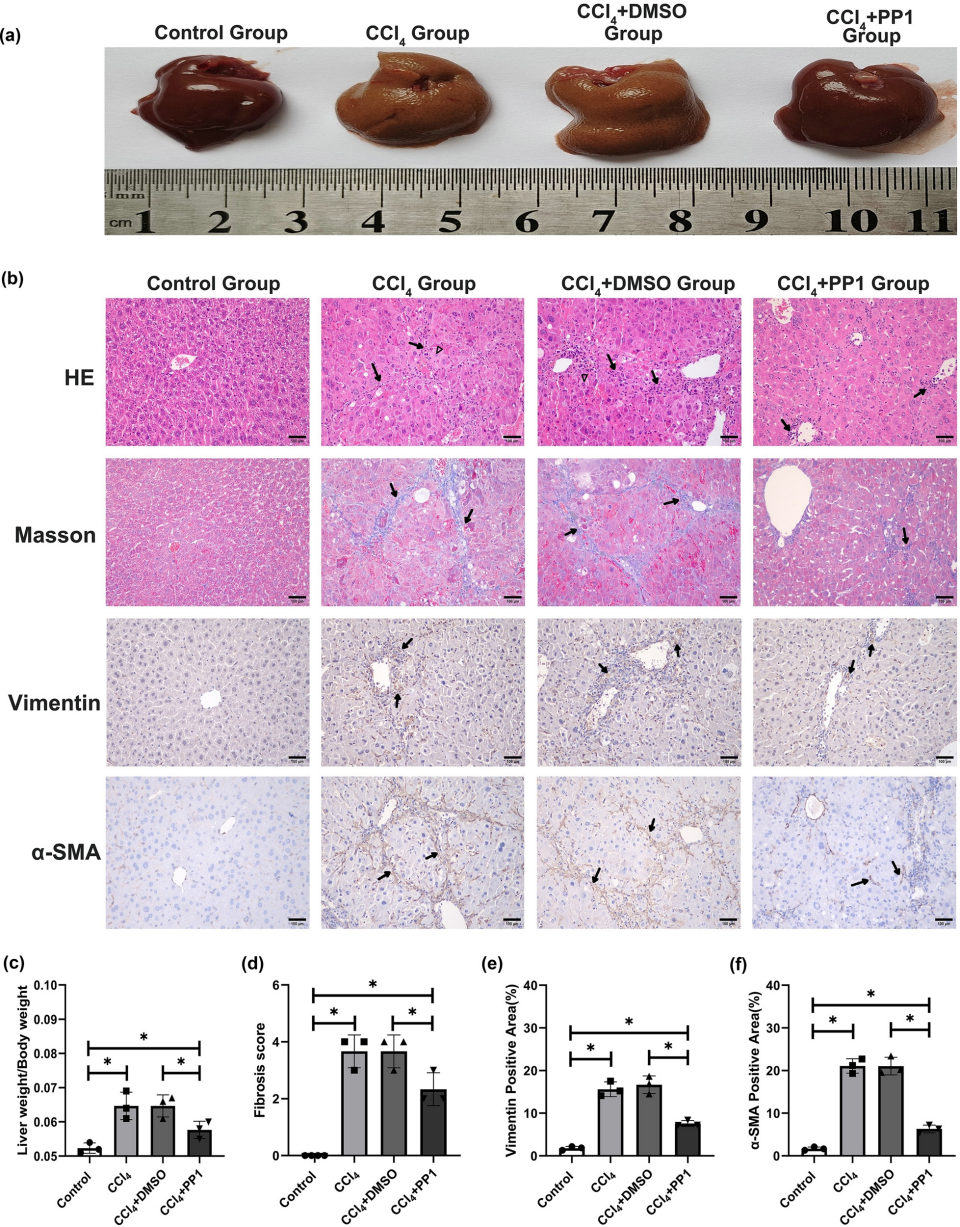
Liver gross morphology, HE and Masson staining, immunostaining of Vimentin and α-SMA, the liver weight/body weight ratio, and fibrosis score in the different groups. (a) Liver gross morphology. (b) HE, Masson staining, and immunostaining of Vimentin and α-SMA. HE, arrows denote inflammatory cell infiltration and triangle symbol denotes hepatocyte edema; Masson, arrows denote collagen deposition; Vimentin, arrows denote Vimentin positivity; α-SMA, arrows denote α-SMA positivity. (c) Liver weight/body weight ratio, * *P* < 0.05. (d) Fibrosis score, * *P* < 0.05. (e) Vimentin-positive area, * *P* < 0.05. (f) α-SMA*-*positive area, * *P* < 0.05.

The results showed that the liver surface in the control group was smooth and soft, and the liver weight/body weight ratio in this group was the lowest. In the CCl_4_ and CCl_4_ + DMSO groups, the liver surface was very rough, with obvious graininess, and the liver weight/body weight ratio was significantly increased (Figure 4a and c). In the CCl_4_ + PP1 group, the liver surface was slightly rough, graininess was significantly reduced, and the liver weight/body weight ratio was significantly lower than that in the CCl_4_ and CCl_4_ + DMSO groups (Figure 4a and c). HE and Masson staining showed that compared with the control group, the CCl_4_ and CCl_4_ + DMSO groups had severe cell edema, inflammatory cell infiltration, fibrous scarring, and collagen deposition, but these changes were significantly alleviated by PP1 treatment (Figure 4b and d). Subsequently, we measured the expression of the liver fibrosis markers Vimentin and α-SMA (Figure 4b). The results showed that Vimentin and α-SMA were not obviously expressed in the control group but were significantly expressed in the CCl_4_ and CCl_4_ + DMSO groups (Figure 4e and f). After PP1 treatment, these two markers were significantly reduced (Figure 4e and f). Therefore, PP1 can significantly reduce CCl_4_-induced liver fibrosis in mice.

### 3.4 Effects on Collagen-I, CTGF, and HIF-1α expression after inhibiting Src activation in fibrotic liver tissue

**Figure 5:**
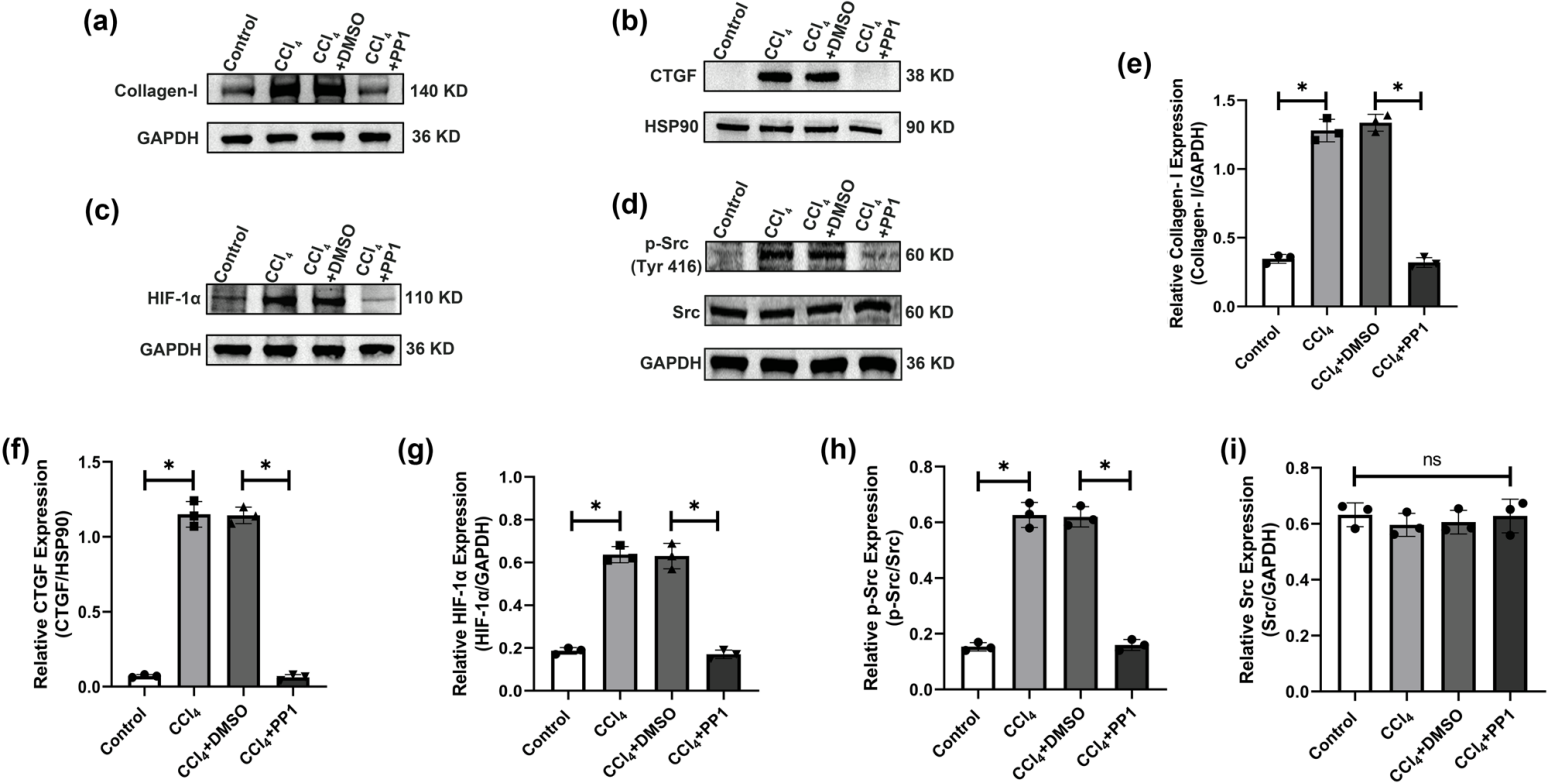
Protein levels of Collagen-I, CTGF, HIF-1α, p-Src, and Src in the different groups. (a–d) Protein levels of Collagen-I, CTGF, HIF-1α, p-Src, and Src. (e–i) Relative expression levels of Collagen-I, CTGF, HIF-1α, p-Src, and Src. *P < 0.05 compared with the other groups.

Western blotting showed that the protein expression levels of Collagen-I, CTGF, HIF-1α, and p-Src (Tyr416) were significantly lower in the control and CCl_4_ + PP1 groups than in the CCl_4_ and CCl_4_ + DMSO groups after inhibiting Src activation (Figure 5a–h). No obvious change was observed in the protein level of Src among the four groups (Figure 5d and i).

### 3.5 Inhibiting Src activation can induce ferroptosis in fibrotic livers

**Figure 6:**
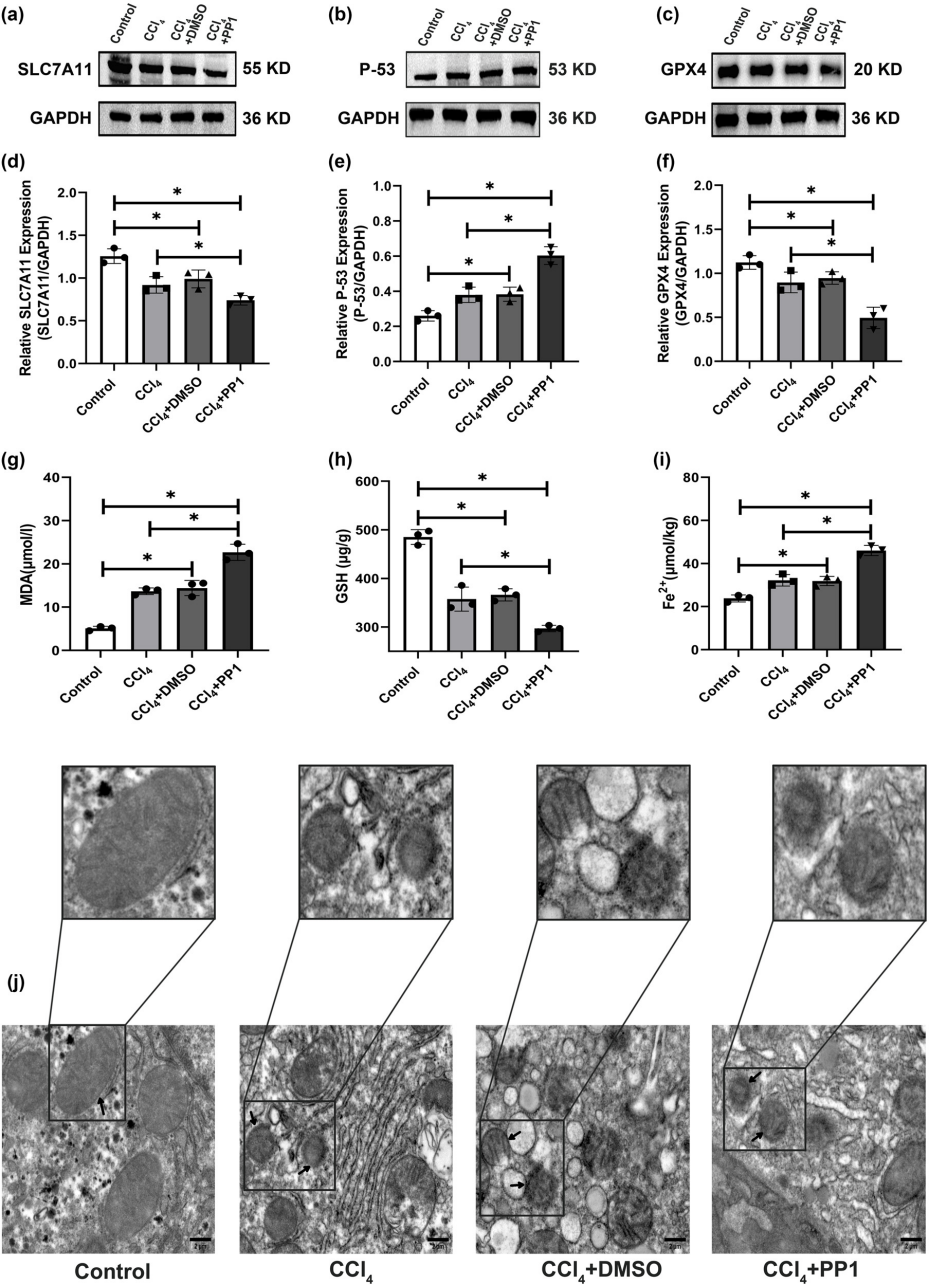
Protein levels of SLC7A11, P-53, and GPX4; the levels of GSH, MDA, and Fe^2+^; and mitochondrial morphology in the different groups. (a–c) Protein levels of SLC7A11, P-53, and GPX4. (d–f) Relative expression levels of SLC7A11, P-53, and GPX4, *P < 0.05. (g–i) GSH, MDA, and Fe^2+^ levels in the different groups, *P < 0.05. (j) Mitochondrial morphology in the different groups. Arrows denote normal mitochondria (control group) and mitochondrial solidification (other groups).

Western blotting showed that the protein levels of the ferroptosis-related genes SLC7A11 and GPX4 were significantly decreased in the CCl_4_, CCl_4_ + DMSO, and CCl_4_ + PP1 groups compared with the control group, and the expression levels of these two proteins were lowest in the CCl_4_ + PP1 group (Figure 6a, c, d, and f). However, the protein levels of the ferroptosis-related gene P-53 in HSC-T6 cells were significantly increased in the CCl_4_, CCl_4_ + DMSO, and CCl_4_ + PP1 groups compared with the control group, and the expression levels of this protein was highest in the CCl_4_ + PP1 group (Figure 6b and e). GSH levels in the CCl_4_, CCl_4_ + DMSO, and CCl_4_ + PP1 groups were lower than those in the control group, and GSH levels in the CCl_4_ + PP1 group were the lowest (Figure 6g); however, compared with those in the control group, the levels of MDA and Fe^2+^ in the CCl_4_, CCl_4_ + DMSO, and CCl_4_ + PP1 groups were increased and were highest in the CCl_4_ + PP1 group (Figure 6h and i). TEM indicated shrunken mitochondria in the CCl_4_, CCl_4_ + DMSO, and CCl_4_ + PP1 groups, but in the normoxia group, mitochondrial morphology was normal (Figure 6j).

The *in vivo* experiments showed that the inhibiting Src activation could promote ferroptosis and alleviate liver fibrosis.

## 4 Discussion

HSCs play an important role in liver fibrosis. After continuous liver injury, HSCs can differentiate into activated MFs, leading to massive, abnormal deposition of ECM components in the liver, resulting in the development of liver fibrosis. Current research suggests that inhibiting HSCs activation or inducing HSCs death are effective ways to prevent liver fibrosis [18]. Ferroptosis is a novel form of programmed cell death that can alleviate liver fibrosis [19].

Src is a member of the tyrosine protein kinase family that plays an important role in the development of renal fibrosis, pulmonary fibrosis, and liver fibrosis [11,20,21]. Hypoxia is an important factor that promotes the development of liver fibrosis and the key to hypoxic damage is HIF-1α. Furthermore, HIF-1α is also important for promoting HSCs activation. In this study, we found that inhibiting Src activation reduced HSCs activation by decreasing HIF-1α expression, and inhibiting HSCs activation is an important way to alleviate liver fibrosis [3,4]. Zou et al. showed that hypoxia promoted ferroptosis in clear cell carcinoma [22], and we found that hypoxia could promote HSCs ferroptosis; furthermore, we observed that ferroptosis in hypoxic liver fibrosis tissues was increased compared with that in normal liver tissues. It is thought that this phenomenon may be associated with increased ROS production by mitochondria under hypoxic condition, which directly promotes lipid peroxidation and thus exacerbates ferroptosis [23,24], but further research is still needed.

Another question is that since hypoxia can promote the occurrence of ferroptosis, and ferroptosis can alleviate liver fibrosis; so does it mean that hypoxia can alleviate liver fibrosis. The answer is no. Although hypoxia can induce ferroptosis in HSCs, the disadvantages of hypoxic damage to liver fibrosis are far greater than the benefits. In addition to promoting the HSCs activation, hypoxia can promote the death of normal liver cells and promote the activation of many pathways related to fibrosis [25,26]. These effects exacerbate liver fibrosis, which has been confirmed by many studies.

Bioinformatics analysis showed that Src and ferroptosis might be associated [27]; however, specific experimental studies are still rare. Huang et al. found that Src homology region 2 domain-containing phosphatase-1 (SHP-1) has a regulatory effect on ferroptosis in hepatocellular carcinoma [28]. In addition, Brown et al. found that in integrin α6β4-depleted human normal mammary epithelial cells (MCF-10A) and breast cancer cells (SUM-159), Src activation is significantly reduced, which promotes ferroptosis in these two types of cells [29]. Our study showed that in liver fibrosis, after Src activation was inhibited, the degree of ferroptosis in both HSCs and fibrotic liver tissue was significantly increased, which could promote the remission of liver fibrosis. This finding suggests that there is a close relationship between Src and ferroptosis, which supports the findings of the bioinformatics analysis [27]. In HSCs, inhibiting Src activation can promote ferroptosis, based on our experimental results, which is considered to be related to the HIF-1α/SLC7A11 pathway [5]. After inhibiting Src activation, HIF-1α expression is inhibited, and GSH synthesis and GPX4 expression can be reduced through the HIF-1α/SLC7A11 pathway, thus promoting ferroptosis in activated HSCs. When HSCs ferroptosis, inflammation in liver tissue is reduced, and the expression of fibrosis indicators, such as α-SMA, Vimentin, Collagen I, and CTGF are decreased; meanwhile, the production of ECM is also reduced. Thus promoting the alleviation of liver fibrosis. However, the specific way in which Src regulates ferroptosis remains unclear, and whether the regulatory effect of Src on ferroptosis in HSCs requires the participation of integrin α6β4 and SHP-1 still needs further study.

**Figure 7:**
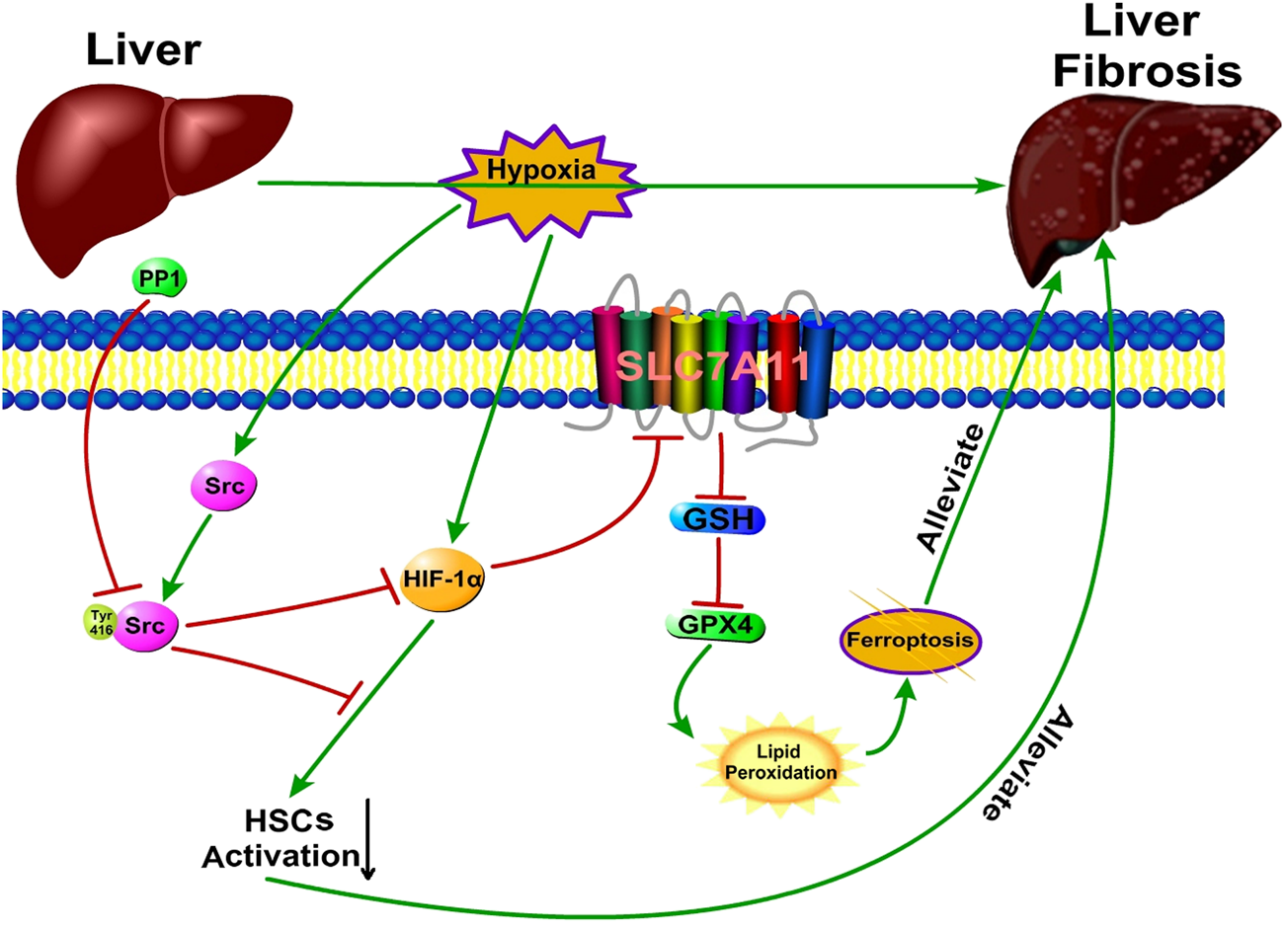
Inhibiting Src activation can induce HSCs ferroptosis and reduce HSCs activation. Ultimately, liver fibrosis can be alleviated.

In summary, our findings indicate that inhibiting Src activation, which increases HSCs ferroptosis, is an important mechanism to alleviate liver fibrosis. These findings will provide new ideas for the therapy of liver fibrosis (Figure 7).

## 5 Conclusion

Liver cirrhosis is a global public health problem. Once liver cirrhosis develops, it is difficult to reverse, and liver fibrosis is considered a reversible pathological process in chronic liver disease that progresses to liver cirrhosis. Therefore, if appropriate therapeutic targets can be found, liver fibrosis may be reversed, which is critical for restoring liver function and improving the quality of life of patients. Src may be such a target, but further research is needed.
